# Plasma-Assisted Atomic Layer Deposition of IrO_2_ for Neuroelectronics

**DOI:** 10.3390/nano13060976

**Published:** 2023-03-08

**Authors:** Valerio Di Palma, Andrea Pianalto, Michele Perego, Graziella Tallarida, Davide Codegoni, Marco Fanciulli

**Affiliations:** 1 Department of Materials Science, University of Milano Bicocca, Via R. Cozzi 55, 20125 Milano, Italy; 2 CNR-IMM Unit of Agrate Brianza, Via C. Olivetti 2, 20864 Agrate Brianza, Italy; 3 STMicroelectronics, Via C. Olivetti 2, 20864 Agrate Brianza, Italy

**Keywords:** pseudocapacitive, atomic layer deposition, IrO_2_, neuroelectronics

## Abstract

In vitro and in vivo stimulation and recording of neuron action potential is currently achieved with microelectrode arrays, either in planar or 3D geometries, adopting different materials and strategies. IrO_2_ is a conductive oxide known for its excellent biocompatibility, good adhesion on different substrates, and charge injection capabilities higher than noble metals. Atomic layer deposition (ALD) allows excellent conformal growth, which can be exploited on 3D nanoelectrode arrays. In this work, we disclose the growth of nanocrystalline rutile IrO_2_ at T = 150 °C adopting a new plasma-assisted ALD (PA-ALD) process. The morphological, structural, physical, chemical, and electrochemical properties of the IrO_2_ thin films are reported. To the best of our knowledge, the electrochemical characterization of the electrode/electrolyte interface in terms of charge injection capacity, charge storage capacity, and double-layer capacitance for IrO_2_ grown by PA-ALD was not reported yet. IrO_2_ grown on PtSi reveals a double-layer capacitance (*C_dl_*) above 300 µF∙cm^−2^, and a charge injection capacity of 0.22 ± 0.01 mC∙cm^−2^ for an electrode of 1.0 cm^2^, confirming IrO_2_ grown by PA-ALD as an excellent material for neuroelectronic applications.

## 1. Introduction

The investigation of neural networks in vitro is fundamental for the understanding of the mechanisms involved in neurological diseases such as Alzheimer’s. In this context, the study of the short- and long-distance interactions between neurons is possible thanks to the development of microelectrode arrays (MEAs), which can be used for the stimulation of neurons and the recording of neuronal signals [1]. MEAs are realized, usually on rigid substrates, either in planar or 3D geometries. The latter approach has been shown to provide a better physical and electrical coupling between the neurons and the electrodes.

The electrical interaction between MEAs and neurons can take place according to two main different charge transfer mechanisms that are determined by the electrical properties of the electrode’s material. The so-called capacitive charge transfer takes place when a dielectric material, which forms a capacitor at the electrode/electrolyte interface, is polarized, and it transfers the polarization to the electrolyte. The other mechanism, commonly occurring for metallic electrodes, consists of a direct, Faradaic transfer of charges between the electrode and the electrolyte [2]. Among the Faradaic charge transfer mechanisms, it is possible to distinguish between the irreversible transfer, generally unwanted, and the reversible transfer. The latter mechanism is generally referred to as pseudocapacitive, since it involves mass exchange at the interface electrode/electrolyte while the stability of the electrode is preserved, as for the capacitive charge transfer [2].

IrO_2_ has been widely applied in the neuroelectronic field because of its pseudocapacitive behavior, along with good stability and non-toxicity [3,4,5]. Nevertheless, the electrical, physical, and chemical properties of the material depend also on the deposition method.

Electrodeposition of IrO_2_ can cause the incorporation of the solvent, generally water, in the film so prepared. The consequence is the formation of a hydrated IrO_2_ layer at the top of the electrode, which is less dense and thus prone to corrosion under working conditions [2]. For the same reason, the use of the so-called activated IrO_2_ (AIROF) poses issues for the long-term stability of the electrode [6,7].

On the other hand, IrO_2_ layers prepared by reactive sputtering for neuroelectronics are often quite thick (about 100 nm or more), posing issues with the amounts of such a rare metal required for electrode fabrication [3,8,9]. In addition, physical deposition methods do not provide conformal growth on 3D substrates with significant aspect ratios. An alternative approach for the deposition of IrO_2_ onto MEAs can be offered by atomic layer deposition (ALD), a thin film deposition method applied in several fields such as micro- and nanoelectronics, spintronics, photovoltaics, electrocatalysis, and neuroelectronics. A key feature of ALD is the self-limiting behavior of the reactions involved, which enables good control of the thickness, high reproducibility, good quality in terms of impurity incorporation, and excellent conformality, i.e., uniformity even onto 3D complex structures [10,11]. ALD also offers the possibility of developing low-temperature processes compatible with flexible substrates such as polydimethyl-siloxane (PDMS).

Only a few reports on the application of IrO_2_ grown by ALD for neuroelectronics have been published so far [5,12]. In addition, the electrochemical characterization of the electrode/electrolyte interface in terms of charge injection capacity, charge storage capacity, and double-layer capacitance for IrO_2_ grown by ALD is still missing. Regarding ALD processes reported in the literature, IrO_2_ is mainly deposited via thermal ALD, using O_2_ or O_3_ as a reactant [13,14,15,16,17], while the use of plasma is still very limited. The use of plasma as a co-reactant can be beneficial in several aspects, besides the advantages already mentioned for ALD. The plasma is generally obtained in the gas phase by an electric field, which accelerates the electrons. These hot electrons then collide with neutral species, generating ions, radicals, and UV photons [18]. The high reactivity of plasma can be beneficial in removing impurities, enabling depositing materials with better electronic properties [18,19,20,21]. Furthermore, the high energy provided to the substrate by the plasma allows it to achieve deposition with a lower thermal budget [18,22,23,24]. Therefore, the use of a plasma-assisted process, instead of thermal ALD, can be beneficial for the integration of the process in the device fabrication, since plasma enables the deposition at lower temperatures (<200 °C). This aspect can be relevant for the fabrication of multi-channel MEAs for neuroelectronics.

There are few reports, to the best of our knowledge, using a plasma-assisted ALD (PA-ALD) process for the preparation of IrO_2_. In 2007 Choi et al. [25] reported PA-ALD of IrO_2_ nanodots using ethylcyclopentadienyl cyclo-hexadiene iridium [(EtCp)Ir(CHD)] dissolved in ethyl cyclo-hexane as a precursor, and a mix of O_2_ and H_2_ plasma as co-reactant. The same combination of precursor and co-reactant was applied in 2014 by Kim et al. [26] for PA-ALD of IrO_2_ nanodots. It is worth mentioning that (EtCp)Ir(CHD) was originally designed and synthesized by Kawano et al. [27] in 2004 as a precursor for the metal–organic chemical vapor deposition (MOCVD) of Ir. Recently, Simon et al. [12] have reported a new PA-ALD of IrO_2_ (on silicon with its native oxide) using O_2_ plasma as a reactant and (Methylcyclopentadienyl) (1,5-cyclooctadiene) Iridium(I) [(MeCp)Ir(COD)].

In this work, we report on a novel PA-ALD approach for the growth of IrO_2_. The process is based on the application of a mix of Ar/O_2_ plasma as a reactant in combination with (EtCp)Ir(CHD) as a precursor. Compared to previous works on PA-ALD of IrO_2_ using (EtCp)Ir(CHD), we did not have to dissolve the precursor in cyclo-hexadiene or feed the plasma source with H_2_ gas. The ALD process is characterized in situ by spectroscopic ellipsometry (SE). In situ SE is a key tool for the characterization of ALD processes, since it is able to monitor thickness changes in the films deposited down to the atomic scale [28,29,30,31]. The determination of the thickness via SE requires modeling the dielectric function of the film. For the specific case of rutile IrO_2_, a Drude–Lorentz oscillator was selected, generally applied for modeling the absorption of light of conductive materials [28,29,30,31].

The chemical, physical, and electrochemical properties of the IrO_2_ thin films have been fully characterized to assess key functionalities relevant to neuroelectronics. Specifically, an extensive electrochemical characterization has been performed, providing key parameters for neuroelectronics, such as the double-layer capacitance, the charge storage capacity, and the charge injection limit. Furthermore, the interpretation of the impedance spectroscopy measurements has been corroborated by the cyclic voltammetry measurements. This last aspect is not very common in the literature on neuroelectronics and can be of broader interest in the electrochemical field.

PtSi has been selected as a conductive substrate for the deposition of IrO_2_. The choice of the substrate is motivated by the planned integration of IrO_2_ onto vertical nanopillar arrays to be used as scalable nanoelectrodes fabricated starting from silicon nanopillars [32].

## 2. Materials and Methods

IrO_2_ deposition was performed using a PICOSUN R-200 Advanced ALD system, equipped with a remote inductively coupled plasma source. The base pressure of the ALD reaction chamber is within the range of 0.2–4.0 hPa. The plasma source operates in the range of 1.9–3.2 MHz, with the plasma power adjustable from minimum of 300 W to maximum of 3000 W. The distance between the plasma source and the sample holder is about 75 cm, in order to reduce any possible damage to the substrate from highly energetic ions. (EtCp)Ir(CHD) (99%), from Strem Chemicals, was kept in a stainless steel cylinder heated to 100 °C, while the line from the cylinder to the deposition chamber was heated to 120 °C. (EtCp)Ir(CHD) precursor should be handled with care, since it causes skin and eye irritation and may cause respiratory irritation. N_2_ gas (99.9999%), used as carrier for the precursor, was flowed at 200 sccm. A mix of Ar gas (99.9999%) and O_2_ gas (99.9999%), flowing at 40 sccm and 190 sccm, respectively, was used to feed the plasma. During the plasma step, the power of the plasma source was set to 2500 W.

The ALD recipe starts with the dosing of (EtCp)Ir(CHD) for 6 s, followed by 15 s of purge. Then, the mixture of Ar/O_2_ is flowed through the plasma source for 1 s in order to stabilize the flow before igniting the plasma for 40 s. Afterward, a purge step of 4 s closes the ALD cycle.

Film Sense FS-1™ ellipsometer system was used for in situ and ex situ characterization of thickness and optical constants of the deposited IrO_2_ layer. The fitting of thickness and optical constants of IrO_2_ thin films was performed using a Drude–Lorentz oscillator.

ALD-prepared IrO_2_ thin films were characterized by X-ray diffraction in grazing incidence mode (GI-XRD), transmission electron microscopy (TEM) and energy dispersive spectroscopy (EDS), time-of-flight secondary ion mass spectrometry (TOF-SIMS), X-ray photoelectron spectroscopy (XPS) and atomic force microscopy (AFM).

Transmission electron microscopy (TEM) techniques were conducted on electron-transparent lamellae obtained by focused ion beam (FIB). The alloy microstructure was observed by bright-field TEM and high-angle annular dark-field STEM (HAADF-STEM), while high spatial resolution chemical analyses were carried out by energy dispersive spectroscopy (EDS). The lamellae were obtained using a Thermofischer Helios G5UX FIB. Low energy milling was used during the final thinning steps to limit heating and ballistic effects of ion irradiation on alloy film. The TEM images were performed with a Thermofischer Themis Z G3 aberration-corrected transmission electron microscope equipped with an FEG electron gun operating at 200 kV acceleration voltage. To minimize the electron beam damage, all the TEM/STEM images and EDS maps were acquired with a low beam current (0.5 nA). The EDS measures were carried out using a Dual-X sensor made of two detectors of 100 mm^2^. The elemental maps were acquired and elaborated by Velox software.

GI-XRD was performed on a Rigaku Smartlab SE equipped with a Cu Ka source (Ka1 = 1.540598) operating at 40 KV and 30 mA. Data were collected under parallel beam conditions, at the angle of incidence of 0.2°, in the range of 20°–50°.

ToF-SIMS profiles were performed using a dual beam IONTOF IV system operating in negative polarity. Sputtering was accomplished using Cs^+^ ions at 1 keV (113 nA) and rastering over a 300 × 300 mm^2^ area. Analysis was performed by means of Ga^+^ ions at 25 keV (1.2 pA) rastering over a 50 × 50 mm^2^ area.

XPS analysis was performed on a PHI5000 Versaprobe III system equipped with a monochromatic Al Ka X-ray source (1486.6 eV) and a concentric hemispherical analyzer with a take-off angle of 45°. Survey spectrum was acquired with a band-pass energy of 280 eV. High-resolution spectra were acquired with a band-pass energy of 55 eV. C 1*s* signal at 284.5 eV was used to correct the binding energy scale.

Film surface morphology was analyzed by atomic force microscopy, using a commercial system (Bruker Dimension Edge). Measurements were carried out in non-contact mode using sharp silicon probes with typical tip radius of 10 nm and resonance frequency of approximately ~320 kHz. Several square scans (1- to 5-micron side) were taken at various surface locations. The acquired data were analyzed by Gwyddion (http://gwyddion.net/; accessed on 7 March 2023) to derive the root mean square (RMS) roughness and the correlation length (L). RMS accounts for the height fluctuations of the surface features, whereas the correlation length is the measure of the length beyond which surface heights are not significantly correlated and it was estimated by the gaussian fitting of the height–height correlation function (see Equations (S1)–(S3), Figures S1 and S2 in the supporting information). The surface parameters reported are the average values over the available data, and the dispersion of these values is reported as the experimental error.

Electrochemical tests were performed using a double-sided-magnetic mount photo-electrochemical cell from Redoxme. Unless differently specified, the electrode area is 1.0 cm^2^. Electrochemical impedance spectroscopy (EIS) was performed using a Zurich Instruments MFIA impedance analyzer, applying a test signal of 100 mV in the frequency range from 0.1 Hz to 100 kHz. EIS characterization of the interface IrO_2_/electrolyte was performed in a two-electrode configuration, with two IrO_2_ samples facing each other in electrical contact through the electrolyte. The phosphate buffer (PBS) used as electrolyte for all the electrochemical characterizations was prepared by diluting 10 mL of PBS 10× purchased from Sigma-Aldrich with 90 mL of deionized water.

Cyclic voltammetry (CV) and voltage transient measurements (VT) were performed using a BioLogic VMP3 multi-channel potentiostat in a three electrodes configuration, i.e., the sample under study as working electrode (WE), an Ag|AgCl wire as reference electrode (RE) and a Pt wire as counter electrode (CE). The Ag|AgCl was selected as reference electrode since it is commonly applied in the field of neuroelectronics [3]; thus, it makes it more straightforward to compare electrochemical characterization performed in this work with the literature.

## 3. Results and Discussion

### 3.1. Plasma-ALD of IrO_2_

**Figure 1:**
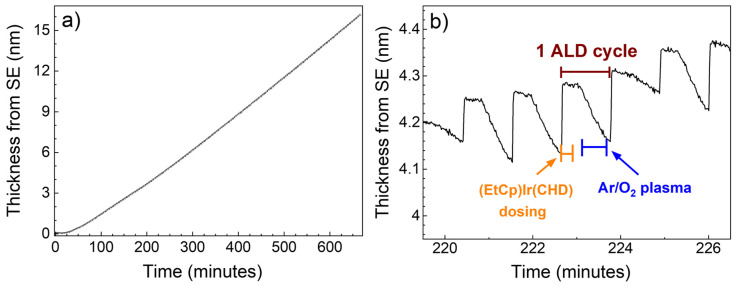
(**a**) Time-resolved in situ spectroscopic ellipsometry measurements during 600 ALD cycles of IrO_2_, at 150 °C onto PtSi. The process exhibits linear growth after a nucleation period of about 50 cycles, corresponding to about 55 min on the time scale. (**b**) Enlargement of the in situ measurement reported in (**a**), showing the characteristic step-like behavior found for the ALD process, with a thickness increase due to the precursor dosing and a subsequent thickness decrease during the plasma dosing due to the removal of ligands.

The in situ characterization of ALD of IrO_2_ (on PtSi) via spectroscopic ellipsometry is shown in Figure 1. From Figure 1a, it is possible to see that, after a nucleation delay of about 50 cycles, the process exhibits linear growth. From the fitting of the experimental data, the growth-per-cycle (GPC) of the process is 0.28 ± 0.01 Å at 150 °C. The value obtained is lower than the value of 0.66 Å reported recently by Simon et al. [12] for PA-ALD of IrO_2_. Nonetheless, the different GPC could be explained by the use of different precursors, i.e., (EtCp)Ir(CHD) in this work and (MeCp)Ir(COD) in the work of Simon et al. [12]. On the other hand, the reports of Choi et al. [25] and Kim et al. [26] on PA-ALD of IrO_2_ focus on the deposition of nanodots; therefore, the GPC value of the process is not specified. From Figure 1b, it is possible to appreciate the thickness changes taking place during each ALD dosing step. The thickness shows a steep increase during the dosing of the (EtCp)Ir(CHD) precursor, due to the adsorption of the precursor on the substrate. Subsequently, during the plasma dosing, the thickness decreases because of the removal of the precursor’s ligands.

**Figure 2:**
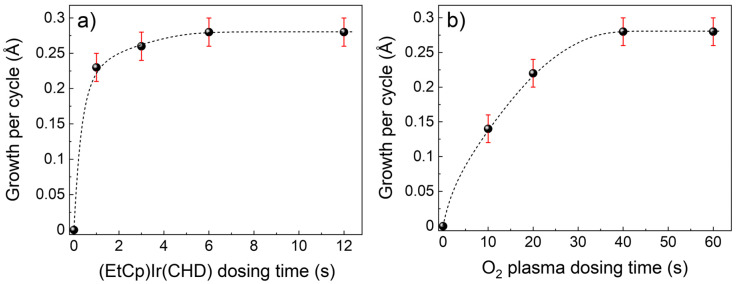
Saturation curves for ALD of IrO_2_. (**a**) GPC as function of the (EtCp)Ir(CHD) dosing time. (**b**) GPC as function of the O_2_ plasma dosing time. Dashed lines are a guide to the eyes.

The saturation of each ALD dosing step was tested by independently changing the dosing times of precursor and co-reactant. Figure 2 shows the GPC as a function of the dosing time for the (EtCp)Ir(CHD) (Figure 2a) and for the plasma (Figure 2b), respectively. The ALD process shows saturation for 6 s of precursor dosing and 40 s of plasma dosing.

### 3.2. Physical and Chemical Characterization of IrO_2_

**Figure 3:**
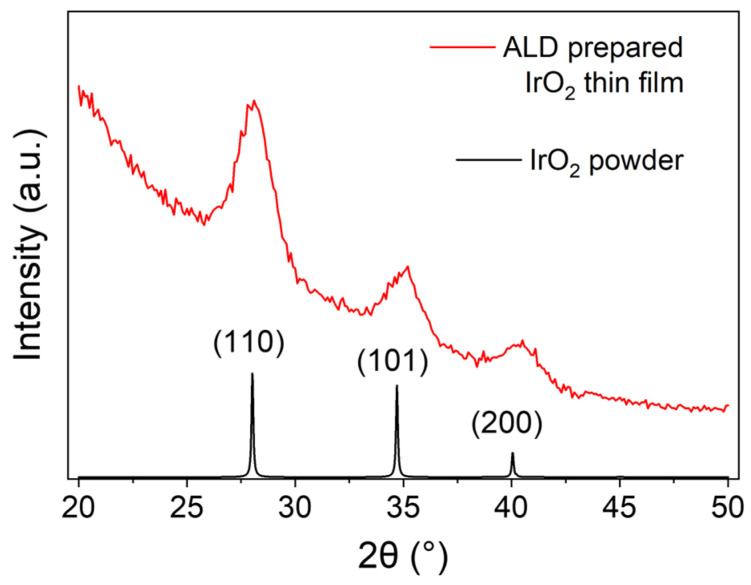
GI-XRD results for IrO_2_ thin film prepared by 600 ALD cycles, corresponding to about 14.8 nm (red line). Angle of incidence ω is 0.2°. The results are compared with the XRD pattern for rutile IrO_2_ powder (black line) calculated with the Mercury Software using the structure deposited on the Cambridge Structural Database as ICSD 640885, deposition number 1759474 [36,37]. The average grain size of IrO_2_, determined by Scherrer’s formula, is 5.3 nm ± 0.4 nm.

The crystallinity of the ALD-prepared IrO_2_ thin films was investigated by grazing incidence X-ray diffraction (GI-XRD). Figure 3 shows that IrO_2_ prepared by ALD at 150 °C reveals the characteristic pattern of IrO_2_ in the rutile phase, with the three peaks corresponding to the <110>, <101>, and the <200> planes, as reported in the literature [33]. The full-width half maximum and the θ of the three peaks were used to determine, by means of Scherrer’s formula, the average grain size of IrO_2_ in the thin film [34,35]. Assuming the shape factor K = 0.9 and knowing the wavelength of the Cu Ka source λ = 1.540598 Å, the average grain size of ALD-prepared IrO_2_ thin film was determined to be 5.3 nm ± 0.4 nm.

**Figure 4:**
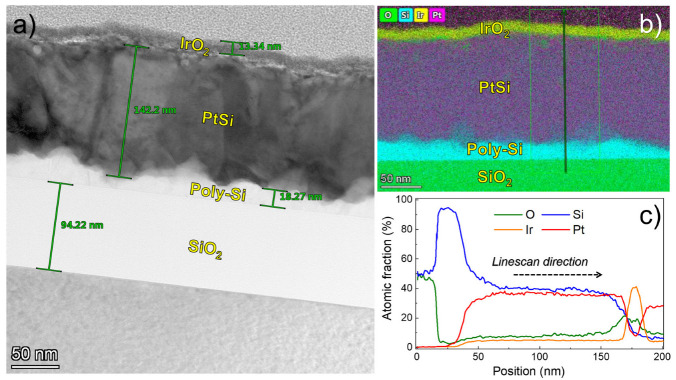
(**a**) TEM cross-section image of IrO_2_ on PtSi. (**b**) EDS mapping of IrO_2_ on PtSi. (**c**) Linescan composition map of IrO_2_ on PtSi. The analysis reveals a continuous and conformal IrO_2_ layer on PtSi. The Pt detected by EDS on the surface is due to the deposition of Pt as an electronic contrast layer during the specimen preparation.

Figure 4 shows the TEM cross-section and TEM-EDS of IrO_2_ grown on PtSi/Poly-Si/SiO_2_/Si. Figure 4a shows a TEM image of the IrO_2_ layer, clearly visible because of the difference in Z-contrast with respect to the PtSi substrate. The layer is homogenous with a thickness of about 13 nm, in line with SE measurements. Figure 4b shows EDS color maps of Ir (yellow), O (green), Pt (purple), and Si (cyan) of the specimen. The Ir-L line, O-K line, Pt-L line, and Si-K line, respectively, were used for the construction of the color maps. The analysis reveals a continuous and conformal IrO_2_ layer on PtSi. Considering the surface roughness of PtSi, the interface between IrO_2_ and PtSi is sharp, with no clear indication of interdiffusion between the two layers. The surface roughness of the underlying substrate has a strong influence on the surface morphology of the IrO_2_ deposited, as suggested by AFM measurements performed on IrO_2_ grown onto Al_2_O_3_ on c-Si (see Figure S3 in the supporting information). Specifically, the RMS of IrO_2_ on Al_2_O_3_ was 0.7 nm ± 0.3 nm, way lower compared to the RMS of IrO_2_ grown onto PtSi (3.5 nm ± 0.3 nm). The EDS line profile in Figure 4c clearly shows Ir and O peaks related to ALD thin film, while the increasing Pt signal on the surface of IrO_2_ is due to the Pt deposited as a contrast layer during the specimen preparation. No further contamination from other elements was detected in the IrO_2_ film within the sensitivity limit of the EDS technique.

**Figure 5:**
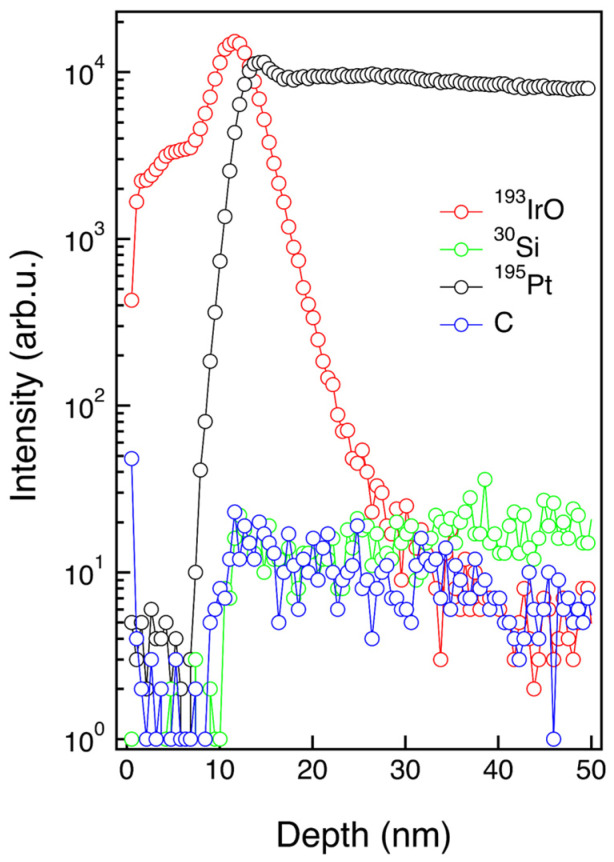
ToF-SIMS depth profiles of the IrO_2_ film deposited on PtSi. ^193^IrO^−^ secondary ion signal indicates the presence of a homogeneous IrO_2_ film with negligible carbon contamination. ^195^Pt^−^ and ^30^Si^−^ signals are reported as markers of the PtSi substrate.

ToF-SIMS depth profile of the ALD-prepared IrO_2_ thin film, shown in Figure 5, confirms the presence of a thin, but homogeneous, IrO_2_ layer on top of the PtSi substrate. ^193^IrO^−^ secondary ion signal and ^195^Pt^−^ and ^30^Si^−^ secondary ion signals are reported as markers of the IrO_2_ film and PtSi substrate, respectively. These signals clearly indicate no diffusion of Pt and Si from the substrate into the IrO_2_ film during the ALD growth. The broadening of the ^193^IrO^−^, ^195^Pt^−^, and ^30^Si^−^ secondary ion signals at the IrO_2_/PtSi interface is fully consistent with the roughness of the PtSi surface that was highlighted by the TEM analysis shown above in Figure 4. No C contaminations were detected in the IrO_2_ film, within the sensitivity limit of the technique, confirming the good quality in terms of impurities for the IrO_2_ thin film prepared by ALD.

**Figure 6:**
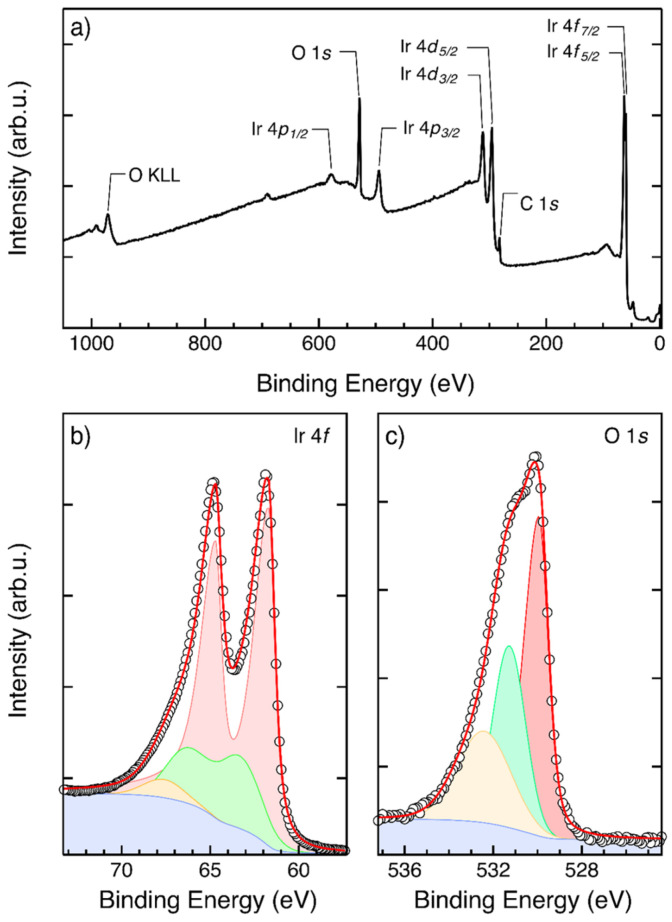
(**a**) Survey spectrum of the IrO_2_ film deposited on top of the PtSi substrate. (**b**) High-resolution Ir 4*f* spectrum (open circles) of IrO_2_ fitted with a doublet of asymmetric main lines (red) and a corresponding doublet of Gaussian functions corresponding to primary shake-up satellites (green). An additional Gaussian function (yellow) is introduced to account for secondary shake-up satellites. Calculated Shirley background (blue) is reported as well. Red line corresponds to the envelope of the fitting functions. (**c**) High-resolution O 1*s* spectrum (open circles) of IrO_2_ fitted with an asymmetric main line (red) and two Gaussian-like lines corresponding to hydroxyl groups (green) and adsorbed water (yellow). Calculated Shirley background (blue) is reported as well. Red line corresponds to the envelope of the fitting functions.

The results of the chemical characterization performed by XPS are reported in Figure 6. Figure 6a shows the survey spectrum of the IrO_2_ film deposited on top of the PtSi substrate. The different core lines in the spectrum correspond to signals from the Ir and O atoms in the IrO_2_ film. No signals from the underlying PtSi substrate were detected, further supporting the idea of a homogeneous IrO_2_ film, perfectly covering the PtSi substrate. Figure 6b,c show high-resolution spectra of the Ir 4*f* and O 1*s* core lines, respectively. Since rutile-type IrO_2_ is a metallic conductor, an asymmetric line shape is expected for the Ir 4*f* core lines. The high-resolution Ir 4*f* spectrum was fitted using a doublet of asymmetric functions with spin–orbit splitting of 3 eV to capture the main 4*f* lines and a doublet of Gaussians at 1 eV higher binding energy to capture the primary shake-up satellites. A secondary satellite of the Ir 4*f*_5/2_ peak at ~3 eV above the main line was introduced to obtain a satisfactory fitting of the experimental data, in agreement with previous results reported in the literature [33]. The position of the Ir 4*f*_7/2_ core line is determined to be 61.7 ± 0.1 eV. The high-resolution O 1*s* spectrum was fitted using asymmetric functions. The position of the O 1*s* core line is found to be 529.9 ± 0.1 eV. Two additional Gaussian functions were introduced to correctly fit the experimental data. These functions account for hydroxyl groups (BE ~ 531.3 eV) and adsorbed water (BE ~ 532.4 eV) on the IrO_2_ surface [38]. The binding energies of the Ir 4*f* and O 1*s* core lines are perfectly consistent with data available in the literature for rutile IrO_2_ [33,38].

**Figure 7:**
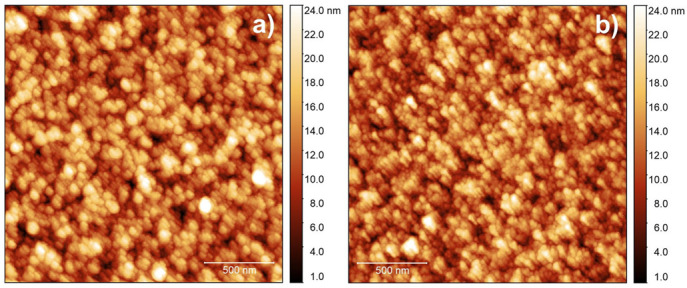
AFM measurement of (**a**) IrO_2_ deposited on PtSi and (**b**) PtSi surface morphology. Both images have scan size of 2 µm × 2 µm and image resolution is 512 × 512 points per line. The thickness of IrO_2_ is about 14.8 nm.

The surface morphology of IrO_2_ films is strictly related to the surface morphology of the supporting substrate. In Figure 7, AFM measurements of the IrO_2_ surface and of the bare PtSi surface are shown. IrO_2_ (Figure 7a) has a marked granular morphology with an RMS roughness of 3.5 ± 0.3 nm and correlation length of 53 ± 3 nm, very similar to that of the PtSi substrate (Figure 7b. RMS roughness: 3.6 ± 0.2 nm; correlation length: 47 ± 3 nm). Thus, in the explored experimental conditions, IrO_2_ film grows conformal to the PtSi substrate, replicating the corresponding surface profile.

### 3.3. Electrochemical Characterization

**Figure 8:**
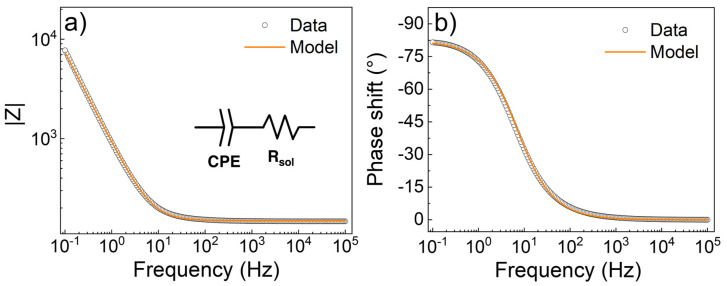
Electrochemical impedance spectroscopy data (black circle) and model (orange line) for the interface IrO_2_/electrolyte. (**a**) Module and (**b**) phase shift of the impedance as function of the frequency. The inset in (**a**) shows the equivalent circuit used for the modeling, i.e., a constant phase element (CPE) for the double layer, in series, with a resistor (*R_sol_*) for the solution bulk.

Electrochemical impedance spectroscopy (EIS) was used to characterize the charge transfer properties of the interface between the ALD-prepared IrO_2_ and the electrolyte. Figure 8 shows the Bode plot, i.e., the module (Figure 8a) and the phase shift (Figure 8b) of the impedance measured as a function of frequency. The equivalent circuit used to fit the data was assembled with a constant phase element (CPE) to model the electrode/electrolyte interface, in series with a resistor (*R_sol_*) used to model the bulk resistivity of the solution. The data analysis was performed using EIS Spectrum Analyser software [39]. The model applied fits the experimental data well, indicating that the charge transfer resistivity, generally used in parallel to the CPE for the modeling of the double layer, tends to infinity. This finding indicates that the ALD-prepared IrO_2_ behaves in solution as an ideally polarized blocking electrode, i.e., no DC is flowing at the electrode/electrolyte interface. The parameters of the CPE, as reported in Table S1 (supporting information), can be used to estimate the double-layer capacitance ($C_{dl}^{EIS}$), according to the surface distribution model, using Equation (1) [40,41]:

(1) $$C_{dl}^{EIS}=Q^{\frac{1}{n}}\cdot R_{sol}{}^{\frac{1-n}{n}}$$

where *Q* and *n* are the parameters defining the impedance of the CPE (see Equation (S4) in supporting information). For the results shown in Figure 8, the double-layer capacitance was calculated to be 301 ± 4 µF∙cm^−2^. This value obtained for ALD-prepared IrO_2_ is very promising, considering that the electrochemically active surface area influences the *C_dl_*, and how in this work, IrO_2_ was deposited onto a relatively flat substrate. As a comparison, a *C_dl_* value of 270 µF∙cm^−2^ has been reported for IrO_2_ electrodeposited onto Ti felt [42].

**Figure 9:**
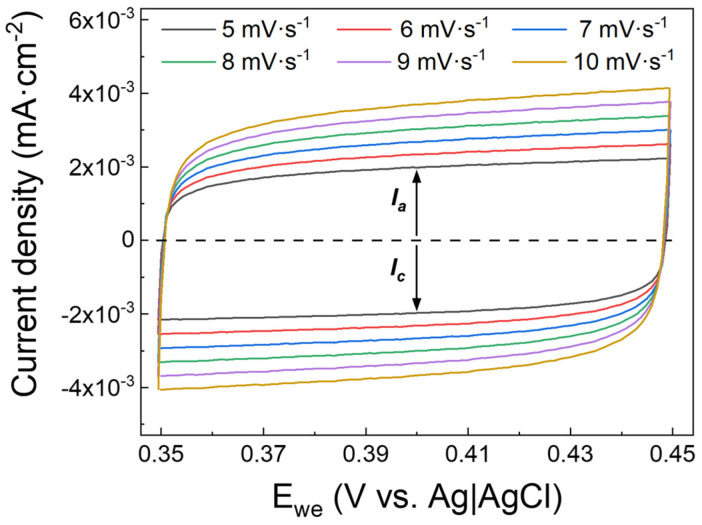
Cyclic voltammograms of ALD prepared IrO_2_ in the range 0.35 V-0.45 V for different scan rates. The values of capacitive cathodic current (I_c_) and capacitive anodic current (I_a_) at 0.40 V are used to determine the double-layer capacitance by the application of Equation (2). CV curves reported are acquired after 50 cycles of stabilization.

In order to corroborate the *C_dl_* value extracted from EIS data, the double-layer capacitance was determined via cyclic voltammetry (CV) as well. A series of CV measurements were carried out, varying the scan rate from 5 mV∙s^−1^ to 10 mV∙s^−1^ in the voltage range between 0.35 V and 0.45 V, where no faradaic processes are supposed to occur [9]. The results reported in Figure 9 show the capacitive cathodic current (I_c_) and the capacitive anodic current (I_a_) as a function of the voltage of the working electrode (E_we_) for different scan rates. The double-layer capacitance can be calculated from the CV measurements via Equation (2) [9]:

(2) $$C_{dl}^{CV}=i_{c/a}\cdot \left(\frac{dE}{dt}\right)$$

where *i_c/a_* is the capacitive current, cathodic or anodic, and *dE/dt* is the scan rate. As reported in Figure S4 (supporting information), the value of $C_{dl}^{CV}$ obtained is about 341 ± 1 µF∙cm^−2^ which is in good agreement with the value obtained by EIS, considering that two different methods, based on different working principles, were used. The high values of *C_dl_* obtained for ALD-prepared IrO_2_ can be explained by considering the good pseudocapacitive behavior of IrO_2_ due to the oxidation/reduction of the Ir(III)/Ir(IV) species at the surface of the electrode [43,44]. It is worth mentioning that it is not common to find, in the literature on neuroelectronics, the comparison between EIS and CV for the characterization of the electrode/electrolyte interface and, more specifically, for the determination of the double-layer capacitance.

**Figure 10:**
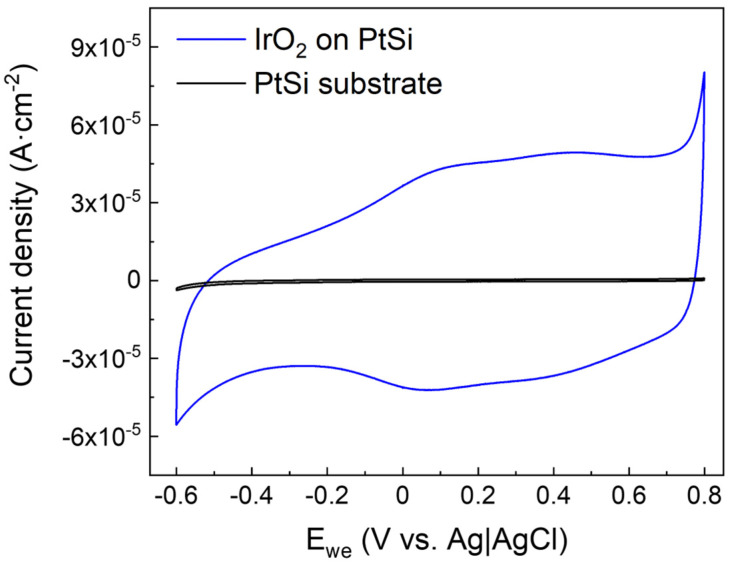
Cyclic voltammograms of ALD-prepared IrO_2_ deposited onto PtSi (blue curve) and of the bare PtSi substrate (black curve). The higher current density of IrO_2_ can be attributed to the pseudocapacitive properties of IrO_2_. Both CV curves reported are acquired after 50 cycles of stabilization, with a scan rate of 50 mV∙s^−1^.

Cyclic voltammetry was then performed in order to determine the charge storage capacity (CSC) of ALD-prepared IrO_2_ in the range of −0.6–0.8 V vs. Ag|AgCl. Figure 10 shows the cyclic voltammogram of IrO_2_ thin film deposited onto PtSi (blue line) compared to the bare PtSi substrate (black line). The current density measured for the IrO_2_/PtSi electrode is much larger than the current density recorded for the PtSi substrate. Since the Faradaic contribution due to the oxidation and reduction of water is negligible below 0.8 V and above −0.6 V, the higher current density of IrO_2_ over the PtSi substrate can be attributed to the pseudocapacitive behavior of IrO_2_, which allows accumulating a larger amount of charge.

The CV curves reported in Figure 10 can be used to calculate the *CSC* by mean of Equation (3) [4,9]:

(3) $$CSC=\frac{1}{v\cdot A}\int_{E_{c}}^{E_{a}}\left|i\right|dE$$

where *E_a_* and *E_c_* are the anodic and cathodic limits of the potential, *i* is the measured current, *v* is the scan rate, and *A* is the surface area of the electrode [4]. For ALD-prepared IrO_2,_ the value of *CSC* was calculated to be 1.9 ± 0.1 mC∙cm^−2^, while the bare PtSi substrate exhibited a *CSC* of (2.4 ± 0.2)·10^−2^ mC∙cm^−2^. The value of *CSC* obtained for ALD-prepared IrO_2_ is slightly lower compared to the non-activated sputtered IrO_2_ reported in the literature (2.8 mC∙cm^−2^) [3]. Nevertheless, a fair comparison should take into account the effective electrochemical surface area. IrO_2_ deposited by reactive sputtering is generally characterized by higher surface roughness, while ALD-prepared thin films are generally smoother since they replicate the surface features of the underneath substrate conformally, as mentioned above in the AFM measurement paragraph.

**Figure 11:**
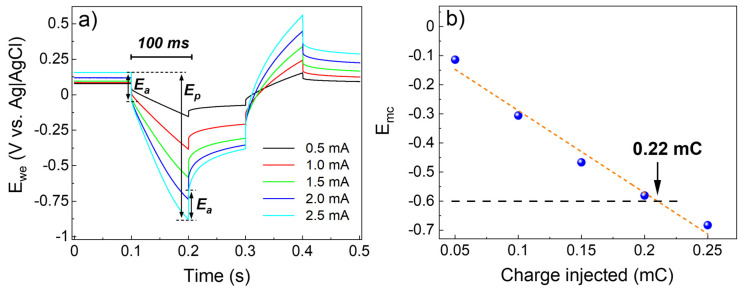
Results of the voltage transient measurements performed on ALD-prepared IrO_2_. (**a**) Shows the voltage of the working electrode (E_we_) as function of time for squared current pulses of 0.5, 1.0, 1.5, 2.0, and 2.5 mA. (**b**) Reports the maximum polarization (E_mc_), calculated by the difference between the peak voltage (E_p_) and the access voltage (E_a_), as function of the charge injected. By linear regression of the data (orange dashed line), it was possible to calculate the charge injection capacity to be 0.22 ± 0.01 mC∙cm^−2^ for E_mc_ = −0.6.

To determine the maximum charge that the IrO_2_ electrode can deliver without overcoming the cathodic limit for the water reduction, voltage transient (VT) measurements were performed. A current squared wave of different amplitudes was forwarded to the sample, recording the voltage (E_we_) over time. The results reported in Figure 11 show the E_we_ as a function of time for values of current amplitude between 0.5 mA and 2.5 mA (Figure 11a). The maximum cathodic polarization (E_mc_) was then calculated by the difference between the voltage peak (E_p_) and the access voltage (E_a_) [3]. Figure 11b shows the E_mc_ as a function of the charge injected. By linear regression of the data, it was possible to calculate that for E_mc_ = −0.6, the charge injected is 0.22 ± 0.01 mC∙cm^−2^. Taking into account that the CIC is inversely proportional to the electrode’s surface area [3], the value obtained can be considered as the lower boundary for ALD-prepared IrO_2_, in view of its application onto microelectrode arrays. We shall also note that CIC values higher than 1.0 mC∙cm^−2^ can be detrimental to neural stimulation because of possible neural damage [45].

## 4. Conclusions

In this work, we disclosed a novel PA-ALD process for the deposition of IrO_2_ using (EtCp)Ir(CHD) as the precursor and a mix of Ar/O_2_ plasma as the reactant. The growth characteristics, the physicochemical as well as the electrochemical properties of IrO_2_ grown by PA-ALD were determined in view of the application in neuroelectronics. The use of the plasma allowed deposition at a relatively low temperature (150 °C), which can be helpful for the integration of the layer on existing devices as well as on flexible substrates. XRD results show the characteristic peaks of the metallic rutile-phase IrO_2_, indicating that the layer so prepared is nanocrystalline. TEM/EDS and ToF-SIMS analysis of ALD-prepared IrO_2_ indicates that the layer is compact and continuous with low carbon contamination. TEM and AFM results highlight the conformality of the layer to the PtSi substrate. XPS results further confirm the presence of the metallic rutile phase of IrO_2_.

Electrochemical characterization showed that IrO_2_ grown by PA-ALD has competitive performances for application in neuroelectronics. Specifically, EIS results revealed a strong capacitive coupling between the IrO_2_ electrode and the electrolyte, with a *C_dl_* of 301 ± 4 µF∙cm^−2^, higher than the value reported in the literature for IrO_2_ electrodeposited on Ti felt [42]. The value of *C_dl_* obtained from CV was about 341 ± 1 µF∙cm^−2^, in general agreement with the value determined with EIS, considering the different working principles of the two methods. To the best of our knowledge, the electrochemical characterization of the electrode/electrolyte interface in terms of charge injection capacity, charge storage capacity, and double-layer capacitance for IrO_2_ grown by PA-ALD was not reported yet. Furthermore, the comparison between EIS and CV results for the determination of the double-layer capacitance is also an element of novelty in the field of neuroelectronics.

The *CSC* of IrO_2_ was calculated in the range of −0.6–0.8 V vs. Ag|AgCl, where the oxidation and reduction reactions of water are negligible. The *CSC* value of 1.9 ± 0.1 mC∙cm^2^ is slightly lower than what is reported for non-activated electrodeposited IrO_2_ (2.8 mC∙cm^2^), although it should be considered that surface roughness has a big role in the electrochemical results. Finally, voltage transient measurements were performed in order to determine the maximum charge that the IrO_2_ electrode can deliver without overcoming the cathodic limit for the water reduction (−0.6 V). The charge injection capacity found for ALD-prepared IrO_2_ was 0.22 ± 0.01 mC∙cm^−2^, in line with the literature [3]. The value obtained can be regarded as a lower limit in view of the integration of IrO_2_ onto MEAs, since the charge injection capacity is inversely proportional to the surface area (1.0 cm^2^ in this work) of the electrodes.

## Data Availability

The data presented in this work are available on request from the corresponding authors.

## Supplementary Materials

The following supporting information can be downloaded at https://www.mdpi.com/article/10.3390/nano13060976/s1. Equation (S1): RMS—root mean square roughness; Equation (S2): height–height correlation function; Equation (S3): gaussian fitting function; Figure S1: AFM of PtSi with the related height–height correlation function; Figure S2: power spectral density function (PSDF) spectra of all the AFM data on IrO_2_ and PtSi; Figure S3: AFM measurement of IrO_2_ deposited by PA-ALD onto Al_2_O_3_ (20 nm) on c-Si; Equation (S4): definition of the CPE impedance; Table S1: parameters of the equivalent circuit obtained from the modeling of the EIS data; Figure S4: plot of the capacitive current vs. scan rate obtained from CV measurements.
