# Supplementary material for: Plasma-Assisted Atomic Layer Deposition of IrO2 for Neuroelectronics
Source: Nanomaterials (Basel). 2023 Mar 8;13(6):976. doi: 10.3390/nano13060976 (PMC10052997; doi:10.3390/nano13060976)
Supplement: Supplementary file 1 [file nanomaterials-13-00976-s001.zip › nanomaterials-2260662-supplementary.pdf]

Supporting information

Plasma-assisted atomic layer deposition of IrO_2 for neuroelectronics

Valerio Di Palma[1,*], Andrea Pianalto[1], Michele Perego[2], Graziella Tallarida[2], Davide Codegoni[3] and Marco Fanciulli[1,*]

1 Department of Materials Science University of Milano Bicocca, Via R. Cozzi 55, Milano, 20125, Italy
2 CNR-IMM Unit of Agrate Brianza, Via C. Olivetti 2, Agrate Brianza, 20864, Italy
3 STMicroelectronics, Via C. Olivetti 2, Agrate Brianza, 20864, Italy
* Correspondence: valerio.dipalma@unimib.it (V.D.P.); marco.fanciulli@unimib.it (M.F.)

AFM

RMS – root mean square roughness is defined as:

$$\sigma = \sqrt{\frac{1}{N}\sum_{n=1}^{N}(z_n - \bar{z})^2} \tag{S1}$$

where z_n are the height values and $\bar{z}$ is the mean value.

Height-Height Correlation Function is:

$$H_x(\tau_x) = \frac{1}{N(M-m)}\sum_{l=1}^{N}\sum_{n=1}^{M-m}\left(z_{n+m,l} - z_{n,l}\right)^2 \tag{S2}$$

where $m = \tau_x/\Delta x$

The Gaussian fitting function is:

$$H_x(\tau_x) = 2\sigma^2\left[1 - exp\left(-{\tau_x^2}/{L^2}\right)\right] \tag{S3}$$

where σ is the root mean square deviation of the heights and L is the correlation length.
As an example, Figure S1 shows the AFM image 5 μm × 5 μm size of PtSi, together with the related H_x function and its Gaussian fit.

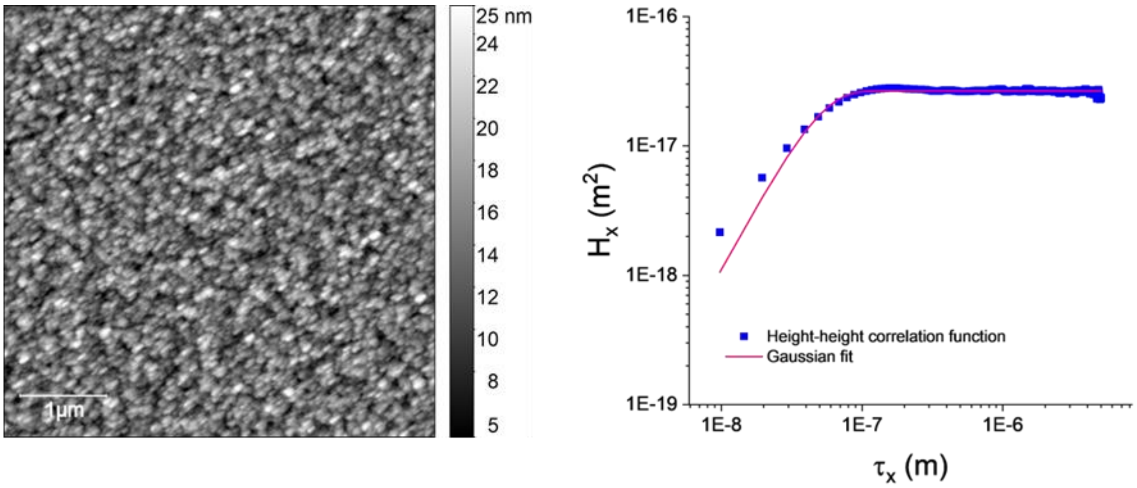

Figure S1. 5 μm × 5 μm AFM image (left) of PtSi and the corresponding height-height correlation function (right). The fitting parameters of the Gaussian fit are σ = 3.5 nm and L = 50 nm.

Figure S2 reports the Power Spectral Density Function (PSDF) spectra of all the AFM data available for IrO_2 (Fig. S2 a) and PtSi (Fig S2 b) and their comparison (Fig S2 c).

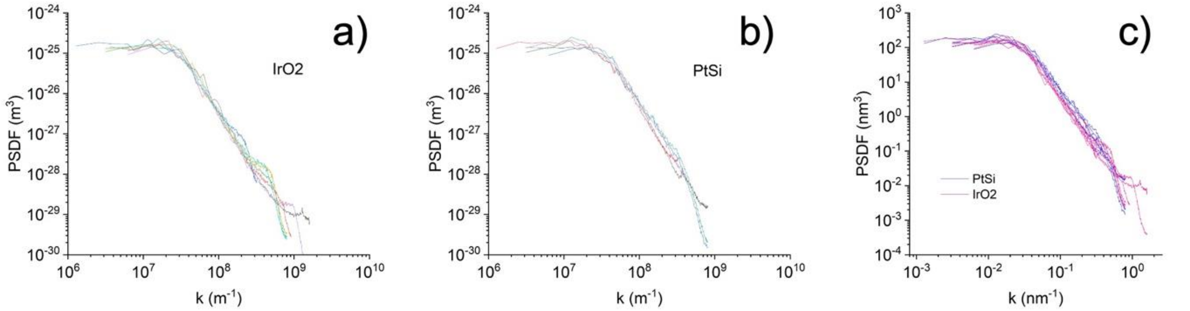

Figure S2. PSDF spectra as derived from the AFM data analysis software Gwyiddion© of all scans taken on a) IrO_2, b) PtSi. PSDF spectra are all collected in c) for comparison. The marked similarity between the PSDF spectra further evidence the conformality of the oxide growth on PtSi. We also note that in our measurements the increase of the scan size does not add relevant information to the PSDF spectra; the curves overlap and the exponential decay beyond the knee at k ~ 0.03 nm^{-1} indicates uniform roughness distribution.

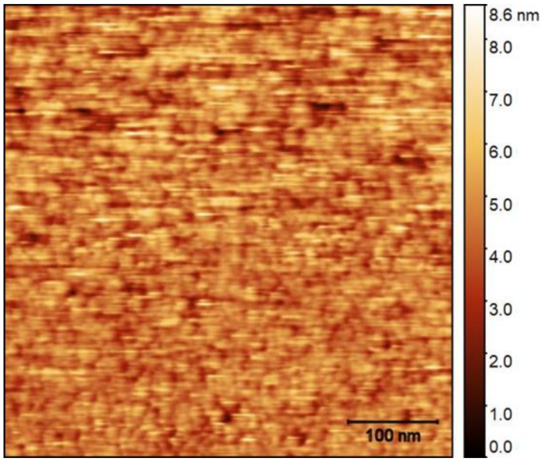

Figure S3. AFM measurement of IrO_2 deposited by PA-ALD onto Al_2O_3 (20 nm) on c-Si. In this case the RMS measured for IrO_2 was 0.7 ± 0.3 nm, confirming that the roughness of IrO_2 grown onto PtSi is actually due to the surface features of the substrate.

EIS

Impedance of CPE is defined as follows, by Equation S4:

$$Z_{CPE} = \frac{1}{Q(j\omega)^n} \tag{S4}$$

with $0 < n < 1$.

Table S1 shows the parameters obtained by the fitting of the EIS data reported in Figure 8 of the manuscript.

Table S1. Values of the parameters obtained from the modeling of EIS data with the equivalent circuit reported in Figure 8 of the manuscript.

Parameter	Value
$\mathbf{R_{sol}}$	148.7 Ω
**Q**	$2.08 \cdot 10^{-4}\ \Omega^{-1} \cdot s^n$
**n**	0.91

CV

Figure S4 shows the capacitive current, obtained from CV measurements reported in Figure 9 of the manuscript, as function of the scan rate. The double layer capacitance was obtained by linear regression of the data points.

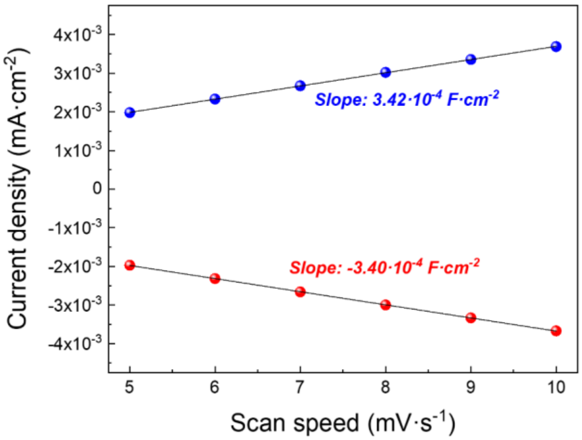

Figure S4. Anodic capacitive current (blue) and cathodic capacitive current (red) as function of the scan rate for the CV measurements reported in Figure 9 of the manuscript. The slope, obtained by linear regression of the data (black lines), gives the value of the double layer capacitance for the interface between ALD prepared IrO_2 and the electrolyte.
